# Melanotic neuroectodermal tumour of infancy: A report of two cases^☆^

**DOI:** 10.1016/j.ijscr.2018.11.004

**Published:** 2018-11-15

**Authors:** Shady A. Moussa, Mohamed ElSayed, Soad Mansour, Fahmy A. Mobarak

**Affiliations:** a Nasser Institute Hospital for Research and Treatment, Cairo, Egypt; b Department of Otorhinolaryngology, Faculty of Medicine, Benha University, Egypt; c Faculty of Dentistry, Princess Nourah Bint Abdulrahman University, Saudi Arabia; d Department of Oral & Maxillofacial Surgery, Faculty of Oral and Dental Medicine, Cairo University, Egypt

**Keywords:** Melanotic neuroectodermal tumour of infancy, Benign lesions, Maxillofacial pathology, Surgical excision

## Abstract

**Introduction:**

Melanotic neuroectodermal tumour of infancy (MNTI) is a benign tumour of infancy, most commonly affecting the head and neck region. First described in 1918, less than 500 cases have been reported in the literature. MNTI is aggressive in nature & has a high rate of recurrence.

**Presentation of cases:**

In this retrospective case series, we report two cases of MNTI that presented at our unit; both cases were managed by wide excision and have been followed up uneventfully for over two years.

**Discussion:**

MNTI has a recurrence rate of up to 20%. Patient’s age can play a significant role in recurrence rate. Although this neural crest tumour is somewhat rare in the literature, there is a consensus with regards to surgical management; the gold standard remains to be wide excision with safety margin. Select cases may benefit from adjuvant and neoadjuvant therapy.

**Conclusion:**

Owing to its locally aggressive nature and high recurrence rate, prompt diagnosis and surgical intervention is advised in cases of MNTI. Further understanding of this tumour is needed on a microscopic level in order to determine clear prognostic factors.

## Highlights

• Shedding light on the aggressive nature and rare incidence of Melanotic Neuroectodermal Tumour of Infancy (MNTI).
• Detailing different clinical and radiographic features of MNTI.
• Outlining the management of MNTI and postoperative follow-up regimen.
• Emphasising the importance of early diagnosis and surgical intervention in Melanotic Neuroectodermal Tumour of Infancy.

## 1 Introduction

We present two cases of Melanotic Neuroectodermal Tumour of Infancy (MNTI), a rare but clinically and histopathologically distinct benign tumour of neural crest origin, found chiefly in new-born infants. It was first described by Krompecher in 1918, who named it congenital melanocarcinoma [[1], [2], [3], [4]].

Historically, the tumour was known by many terms, including melanotic epithelial odontoma, pigmented teratoma, retinal anlage tumour and melanocytoma. The current designation of melanotic neuroectodermal tumour of infancy was adopted in 1992 by the World Health Organisation (WHO) classification of odontogenic tumours [5,6].

Management of this rapid growing, locally aggressive tumour entails complete excision with a safety margin of 0.5–1 cm. Adjuvant chemotherapy and, to a lesser extent, radiotherapy can be utilised in conjunction with surgery. Larger lesions that cannot be resected primarily may benefit from neoadjuvant chemotherapy [1,2,7,8].

In this retrospective case series, we report two cases of MNTI that presented at our unit within a short period in 2015. This case series aims to shed a light on this rarely-reported lesion and the prompt surgical intervention which was curative in both patients under our care.

This case series has been reported in line with the Preferred Reporting of Case Series In Surgery (PROCESS) criteria [9].

## 2 Case 1

**Fig. 1:**
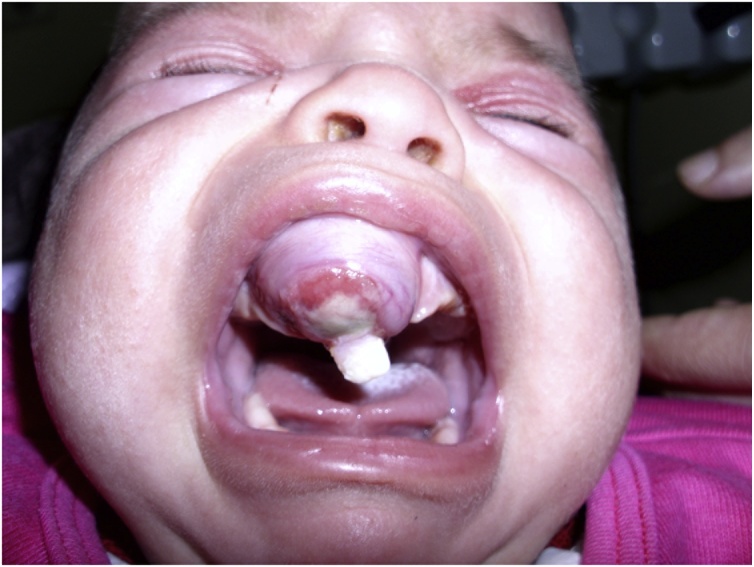
Patient 1 preoperatively.

Patient one, a 3-month-old female patient, presented in March 2015. Her parents had noticed a rapidly growing maxillary swelling during the previous month. The patient’s medical history was insignificant. On examination, a firm swelling measuring 3 × 4 cm was detected on the anterior maxilla. The overlying mucosa was ulcerated in the middle, with a deciduous incisor exfoliating through the lesion (Fig. 1).

**Fig. 2:**
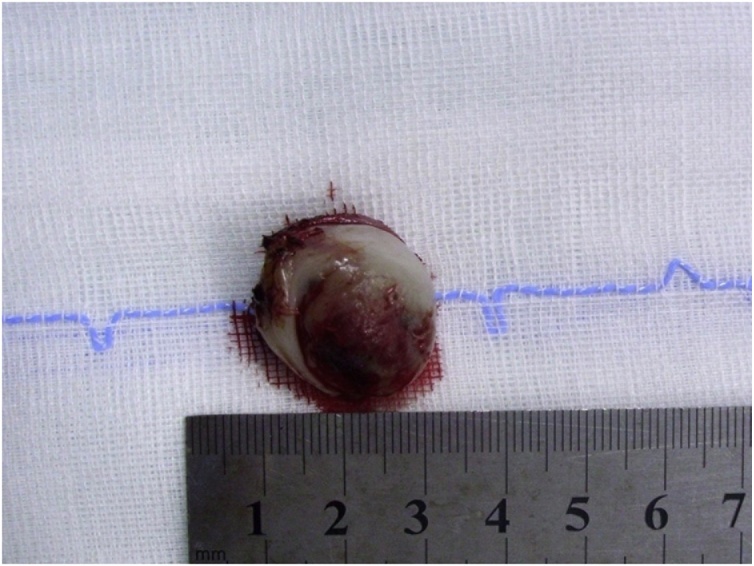
Excised lesion from Patient 1 following first surgery.

**Fig. 3:**
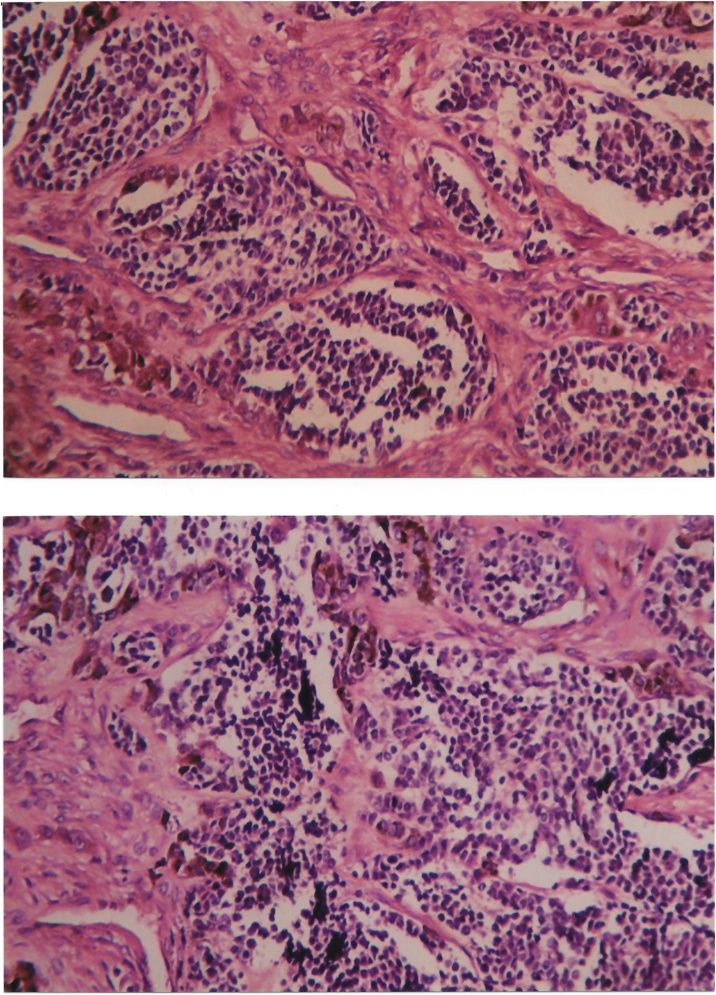
Histopathological specimen showing clusters of lymphocytes with intervening fibrous stroma containing intra-cytoplasmic brown pigments.

Multislice Computed Tomography (CT) revealed a well-defined osteolytic lesion encroaching on the right anterior maxillary wall. Incisional biopsy, performed by a team led by author FAM, confirmed a diagnosis of melanotic neuroectodermal tumour of infancy. Subsequently, a second surgery was performed in April 2015, with tumour excision via a transoral approach (Fig. 2, Fig. 3).

**Fig. 4:**
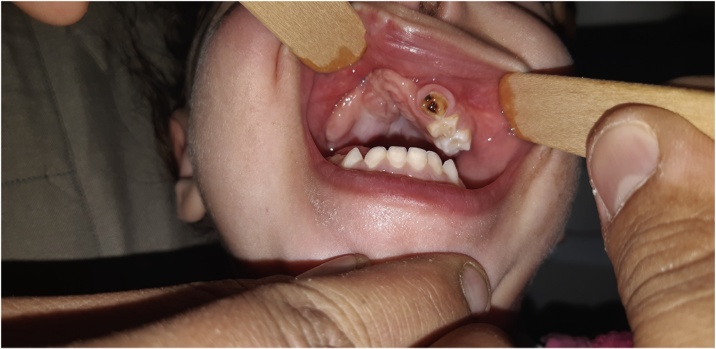
Three-year postoperative photograph of Patient 1 showing satisfactory healing.

**Fig. 5:**
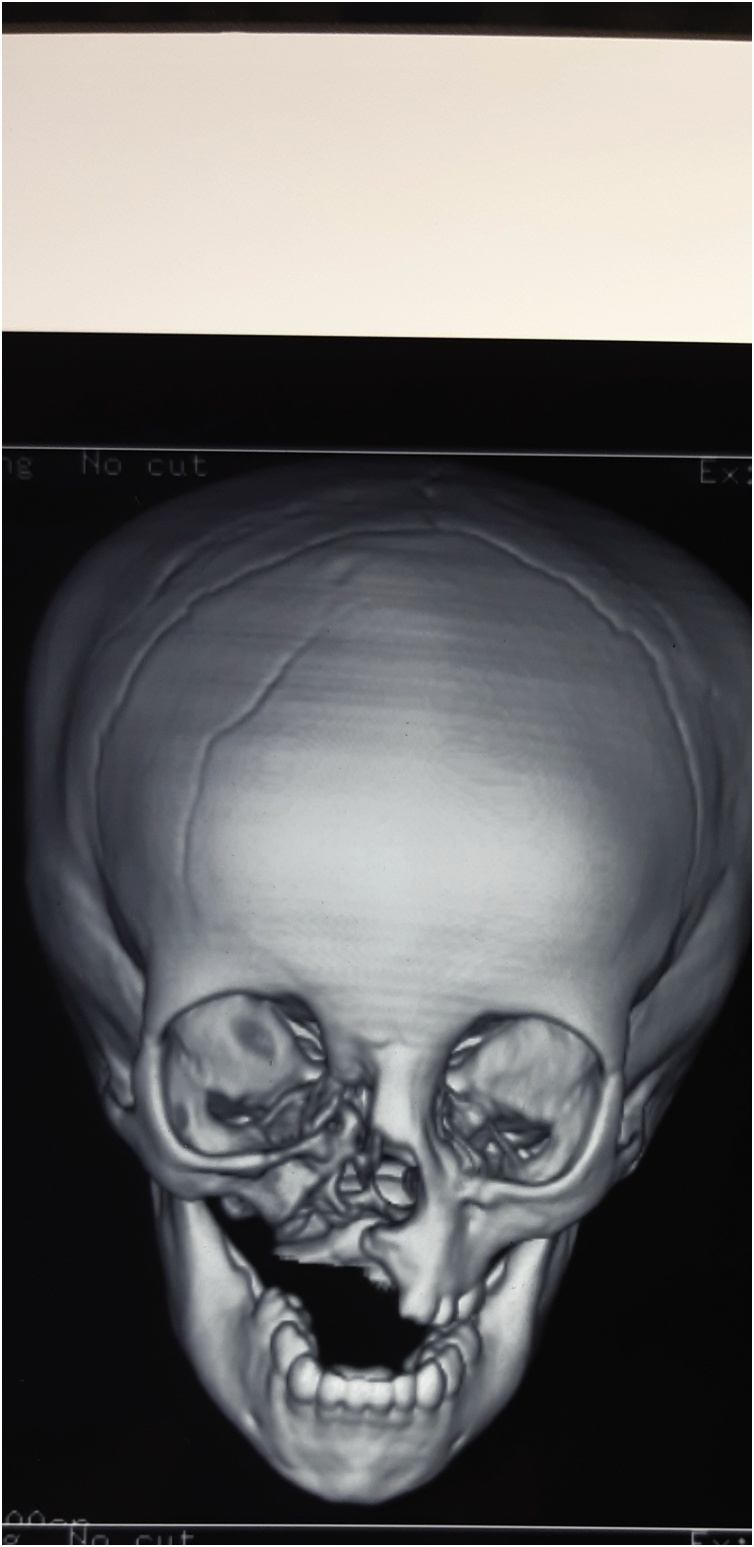
Three-year postoperative 3D reconstruction CT of Patient 1.

Possibly due to the conservative nature of the surgical excision and/or tumour seeding, a recurrence of the lesion occurred four months later in August 2015. Via a Weber Ferguson approach, a right subtotal maxillectomy was performed to resect the recurrent tumour with a safety margin of 1 cm. Histopathology affirmed the diagnosis of MNTI. The patient’s subsequent recovery was uneventful; she has been followed up for over three years, with no incidence of recurrence clinically or radiographically (Fig. 4, Fig. 5).

## 3 Case 2

The second patient was a 4-month-old female infant, who presented to our unit in December 2015 after her parents noticed a progressively growing left maxillary mass of gradual onset.

**Fig. 6:**
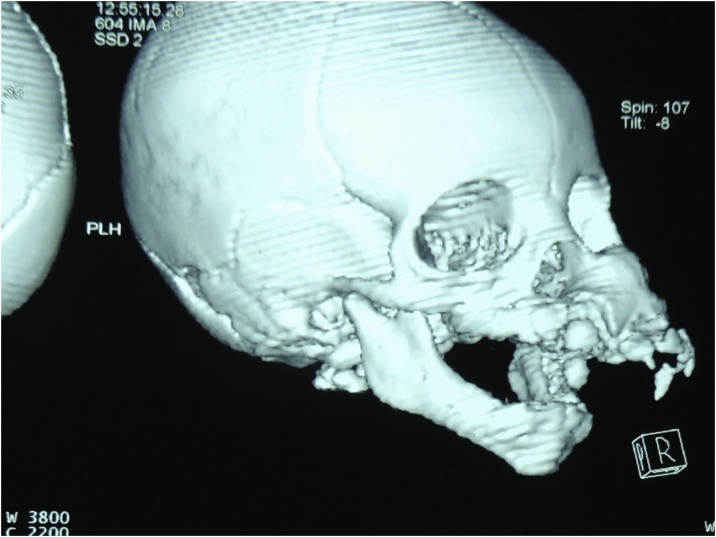
Preoperative 3D reconstruction CT of patient 2, showed marked expansion of the Left maxilla.

**Fig. 7:**
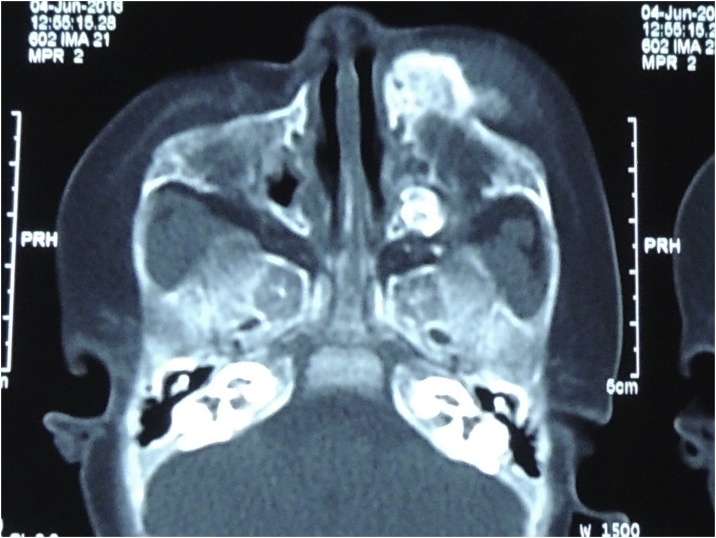
Preoperative Computed Tomography (Axial cut) showing a poorly-defined radiopaque lesion of the left maxilla.

On examination, a well-defined firm mass of the left maxilla was detected. The lesion was roughly 4 × 5 cm in size and smooth in texture, with an ulcer measuring 1 × 1 cm located at the lesion’s surface. Computed Tomography revealed an expansile lesion of the left maxilla with poorly-defined margins (Fig. 6, Fig. 7).

An incisional biopsy revealed a diagnosis of Melanotic Neuroectodermal Tumour of Infancy. Histologically, the specimen showed groups of round cells with abundant cytoplasm and pale nuclei, surrounding nests of neuroblast-like cells possessing scant or fibrillar cytoplasm. Immunohistochemistry confirmed the specimen was positive for both HMB45 and Synaptophysin.

A thorough work-up was subsequently performed, including Computed Tomography of the chest, abdomen and pelvis to rule out any metastasis; this was negative for any tumor spread.

**Fig. 8:**
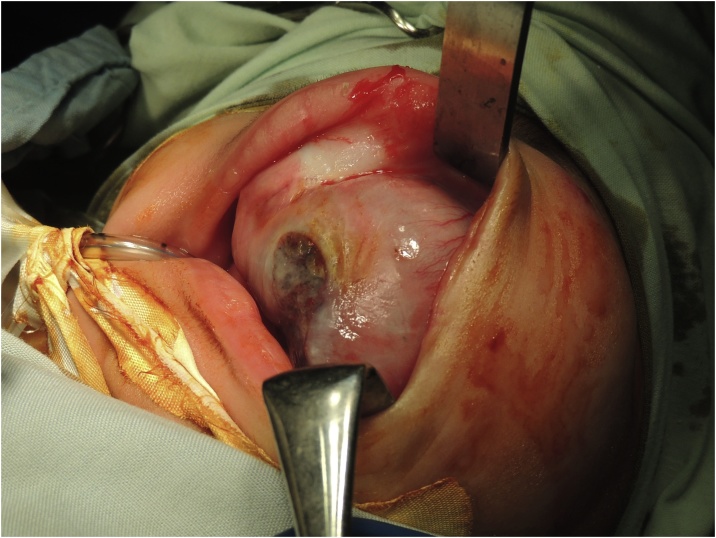
Intraoperative image of Patient 2 prior to tumour excision.

**Fig. 9:**
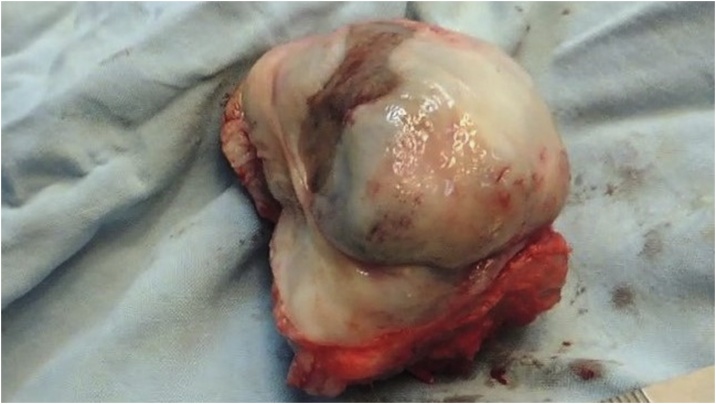
Excised lesion from Patient 2.

Via a Weber Ferguson approach, a surgical team headed by author ME performed a left subtotal maxillectomy and the tumour was excised with a safety margin of 1 cm. The surgical defect was closed primarily with the use of a buccal fat pad and no reconstructive procedure was taken (Fig. 8, Fig. 9).

**Fig. 10:**
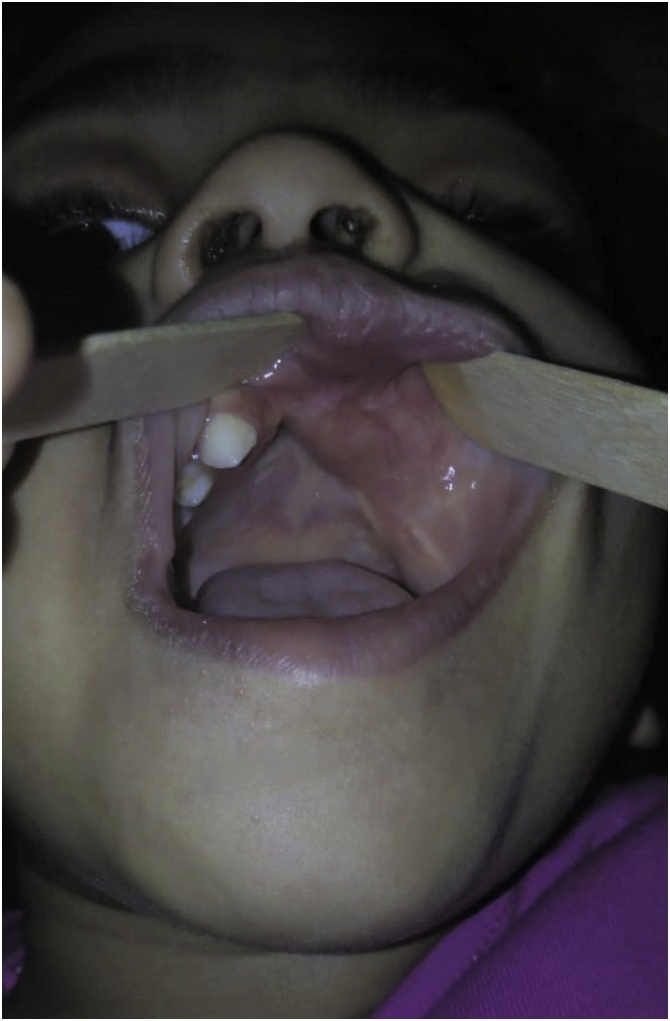
Clinical image of Patient 2 two years postoperatively.

A follow-up CT was taken 18 months postoperatively, with no recurrence detected. Accordingly, a minor residual soft tissue defect in the left premaxilla was closed via a local flap in July 2017. The patient has been followed up for over two years following the MNTI excision, with no signs of recurrence clinically or radiographically (Fig. 10).

## 4 Discussion

First described as congenital melanocarcinoma by Krompecher in 1918, melanotic neuroectodermal tumour of infancy is a tumour of neural crest origin. The presence of vanillylmandellic acid is pathognomonic for the neural crest component. Up to 1990, roughly 200 cases had been reported in the literature; that has increased to roughly 486 cases reported to date [1,3,4].

MNTI most commonly occurs within the first year of life, with a peak incidence between two to six months of age; with 80% of patients under 6 months old. The lesion has a slight male predilection and an affinity for the head and neck, most commonly the maxilla, with 92.8% of cases occurring in the craniofacial region. MNTI may be found both centrally and peripherally; most commonly arising in the anterior maxilla (69%), skull (11%), mandible (6%), the upper limb, thigh and epididymis [[6], [7], [8]].

Radiographically, MNTI often manifests as an expansile lesion with hazy or ill-defined margins, often causing displacement of any adjacent teeth. The tumour is frequently radiolucent but may also present as either a radiopaque or mixed radiolucent/radiopaque lesion. Owing to their melanin content, soft tissue components of the lesion may appear hyperdense on Computed Tomography. Magnetic resonance imaging with gadolinium contrast is also helpful in the imaging of the tumour, appearing isointense on T1-weighted images, with intratumoural melanin appearing hyperdense [5,6,10,11].

Histologically, this biphasic tumour is characterised by large, polygonal epithelioid cells with intracellular brown granular pigmentation and smaller neuroblast-like cells in a stroma of fibrous tissue containing fibroblasts and blood vessels. Immunohistochemically, cytokeratin, HMB45 and vimentin are positive in the larger epithelioid cells. Synaptophysin and enolase are positive in the neuroblast-like cells [2,5,7].

Differential diagnosis for MNTI includes other small round cell neoplasms such as neuroblastoma, rhabdomyosarcoma, peripheral neuroepithelioma, Ewing’s Sarcoma, myeloid sarcoma, melanoma and lymphoma [1,3,8].

This locally aggressive tumour’s nature initially led authors to believe it was malignant in nature, whereas it exhibits malignant transformation in 6.5%–6.97% of cases. Metastatic MNTI most frequently spreads to regional lymph nodes and is often fatal [1,7].

Although Melanotic Neuroectodermal Tumour of Infancy is somewhat rare in the literature, most authors agree with regards to surgical management; the gold standard is wide excision with clear margins. A safety margin of 0.5–1 cm has been reported to suffice in extensive lesions. However, it must be mentioned that a systematic review by Rachidi et al found no difference in recurrence rates in patients treated by curettage only and patients who underwent resection. Adjuvant and neoadjuvant therapy may be utilised in cases of recurrence, cases exhibiting malignant transformation and larger lesions unamenable to primary surgical intervention [1,7,8,12,13].

The tumour has a frequently reported recurrence rate of up to 20%, but recurrence rates of up to 60% have been cited. A systematic review by Rachidi et al showed that the recurrence rate for tumours seen in patients younger than two months of age is quadruple that of tumours seen in patients 4.5 months of age and older. Recurrence is also reported to occur more commonly within four weeks of surgery. This may be either to incomplete surgical excision, tumour dissemination during surgery or multicentricity. Kruse-Lösler et al reported the relative risk for recurrence appeared to be highest in tumours occurring in the mandible, which showed recurrence rates of up to 33%, compared to 19.3% in maxillary lesions [1,7,[12], [13], [14]].

Different prognostic factors have been hypothesised regarding MNTI. Higashi et al reported that the percentage of smaller neuroblast-like cells in the neoplasm is directly proportional to tumour aggressiveness. Accordingly, MNTI with abundant neuroblast-like cells are rapid-growing, while tumours with predominately large cells are slow-growing. Another potential prognostic factor for tumour aggressiveness is the neuroblast-like cell population staining positive for Ki-67 & CD99 Immunohistochemically [2,13,15].

Due to this high rate of local recurrence, numerous authors emphasise the importance of monthly follow-up appointments for the first year postoperatively, complemented with an annual MRI of the tumour site [1,13,14].

## 5 Conclusion

We were exposed to two cases of MNTI within a relatively short period of time at our unit. Both patients were female, and the principles of immediate intervention and total excision with a safety margin were adhered to in both cases.

One patient exhibited recurrence following initial surgical excision, most likely attributed to incomplete surgical excision. Following a subsequent resection with a 1 cm safety margin, she has been followed up for over three years with no signs of recurrence. Despite the postoperative follow-up period for both patients being relatively short, our management of both cases has yielded satisfactory results insofar.

Ideally, a longer follow-up period is required to reach more concrete conclusions. Further understanding of this tumour on a microscopic level is also needed to determine clear, unequivocal prognostic factors for melanotic neuroectodermal tumour of infancy.

A potential risk faced when treating locally aggressive lesions such as MNTI is treating it as a malignant lesion; resulting in overly aggressive resection of the lesion. This can subsequently limit the patient’s postoperative reconstruction options and, ultimately, quality of life. This emphasises the importance of finding a balance between surgical excision and preserving a healthy tissue bed for future rehabilitation.

## Conflict of interest

No conflict of interest to report.

## Sources of funding

No funding bodies or sponsors were involved in this study.

## Ethical approval

Ethical Approval was obtained from the Department of Oral & Maxilloacial Surgery, Nasser Institute Hospital.

## Consent

**Patient1:** Written informed consent was obtained from the patient’s guardian for publication of this case series.

**Patient 2:** Written informed consent was obtained from the patient’s guardian for publication of this case series.

## Author contribution

• Mr Shady A. Moussa ***BDS MSc MFDS RCSEd*(Corresponding Author, Study Design, Data Collection, Writing the Paper)**.
• Professor Dr Fahmy Abdelaal Mobarak BDS MDS PhD **(Lead Clinician, Study Design, Data Analysis, Data Collection)**.
• Dr Mohamed ElSayed MBBCh BDS MSC MD **(Data Collection, Data Analysis)**.
• Professor Dr Soad Mansour BDS MDS PhD **(Study Design, Data Analysis)**.

## Registration of research studies

Research Registry UIN: researchregistry4251.

https://www.researchregistry.com/browse-the-registry.

## Guarantor

Shady Abdelsalam Moussa, Corresponding Author.

## Provenance and peer review

Not commissioned, externally peer reviewed.
